# Functional analysis of *PagERF021* gene in salt stress tolerance in *Populus alba × P. glandulosa*

**DOI:** 10.1002/tpg2.20521

**Published:** 2024-10-16

**Authors:** Gaofeng Fan, Yuan Gao, Xinyue Wu, Yingying Yu, Wenjing Yao, Jiahui Jiang, Huanzhen Liu, Tingbo Jiang

**Affiliations:** ^1^ State Key Laboratory of Tree Genetics and Breeding Northeast Forestry University Harbin China; ^2^ Co‐Innovation Center for Sustainable Forestry in Southern China/Bamboo Research Institute Nanjing Forestry University Nanjing China

## Abstract

Poplar trees are crucial for timber and greening, but high levels of salt in the soil have severely limited the yield of poplar. Ethylene response factor (ERF) transcription factors play an important role in growth, development, and stress response in eukaryotes. Our study focused on the *PagERF021* gene from *Populus alba* × *P. glandulosa*, which was significantly upregulated in various tissues under salt stress [Correction added on October 4, 2024, after first online publication: “ETS2 reporter factor” is changed to “Ethylene response factor”.]. Both the tissue‐specific expression pattern and β‐glucuronidase (GUS) staining of pro*PagERF021*‐GUS plants indicated that this gene was predominantly expressed in the roots and stems. The subcellular localization showed that the protein was only localized in the nucleus. The yeast assay demonstrated that this protein had transcriptional activation activity at its C‐terminal and could specifically binding to the MYB‐core *cis*‐element. The overexpression of *PagERF021* gene could scavenge the accumulation of reactive oxygen species and reduce the degree of cellular membrane damage, indicating that this gene enhanced the salt tolerance of poplars. This finding will provide a feasible insight for future research into the regulatory mechanisms of *ERF* genes in resisting to abiotic stress and the development of new stress‐resistant varieties in plants.

## Core Ideas

- *PagERF021*, a DREB gene, could be induced by salt stress and predominantly expressed in the roots and stems.
- The PagERF021 protein was a nuclear localization protein with transcriptional activation activity.
- *PagERF021* gene could enhance salt tolerance by scavenging ROS and reducing cell membrane damage in poplar.

**Abbreviations**
ABA: abscisic acid
AbA: Aureobasidin A
CTAB: cetrimonium bromide
ERF: Ethylene response factor
GA: gibberellin
GFP: green fluorescent protein
GUS: β‐glucuronidase
MDA: malondialdehyde
NAA: α‐naphthaleneacetic acid
qRT‐PCR: quantitative real‐time polymerase chain reaction
ROS: reactive oxygen species
TF: transcription factor
6BA: 6‐Benzylaminopurine

## 1 INTRODUCTION

High salt is one of the important abiotic stresses that generate a large amount of metal ions (Na^+^), causing serious negative impacts on plant growth, development, and yield by disrupting cell homeostasis and exacerbating plasma membrane damage (Hasegawa et al., 2000; Yang & Guo, 2018). In response to adverse abiotic stresses, plants have developed various adaptive mechanisms at molecular, cellular, physiological, and biochemical levels (Danquah et al., 2014; Ji et al., 2013). At molecular level, plants have been able to protect themself by altering the expression of stress‐related genes, functional proteins, and enzymes through signal transduction (Yang & Guo, 2018). As the initiator of a series of functional genes, transcription factors (TFs) play a vital role in stress response processes, which regulate the expression of special target genes by binding to the corresponding *cis*‐acting elements in promoter regions (Gujjar et al., 2014; Yoon et al., 2020). Although increasing important TFs that participate in stress regulatory pathways have been reported, many unknown TFs remain to be further explored in plants.

Ethylene response factor (ERF) family is one of the largest TF families in plant kingdom (Agarwal et al., 2006; Xie et al., 2022) [Correction added on October 4, 2024, after first online publication: “ETS2 reporter factor” is changed to “Ethylene response factor”.]. ERF TFs typically possess one or more AP2/ERF domains, each consisting of approximately 60 amino acid residues. These domains include a 19–22 amino acid residue YRG motif and an approximately 42 amino acid residue RADY motif (Okamuro et al., 1997). The members of this family can be categorized into four subfamilies: ERF, AP2, DREB (the dehydration responsive element binding), and RAV (Zhuang et al., 2008; Zhuang & Zhu, 2014). Among them, DREB TFs specifically interact with C‐repeat/DRE (A/GCCGAC) element, playing a pivotal role in stress tolerance by regulating the expression of stress‐responsive genes (Sakuma et al., 2002). All members of this family feature a single AP2 domain, with conserved amino acid residues at positions 14 (Val) and 19 (Glu). These residues are recognized as crucial for binding to DRE (dehydration‐responsive element) motifs (Sakuma et al., 2002; Y. Wu et al., 2022).

The DREB TFs play a significant role in regulating plant growth and development. For example, *DREB2C* was capable of interacting with *ABF2* to co‐activate the expression of abscisic acid (ABA)‐related genes in *Arabidopsis*. The overexpression of *DREB2C* influenced the sensitivity of plants to ABA, which was a key hormone involved in stress responses (S. J. Lee et al., 2010). *TaRAP2.1* functioned a DREB transcriptional repressor and overexpression of the gene barley resulted in dwarfism in wheat (Amalraj et al., 2016) [Correction added on October 4, 2024, after first online publication: “results” is change to “resulted”.]. *OsDREB2B* was capable of reducing gibberellin (GA) content by modulating the expression of genes involved in GA metabolism. This regulation was mediated through the interaction between *OsAP2‐39* and *OsWRKY21*. Consequently, *OsDREB2B* negatively regulated the plant height in rice by influencing GA levels (Ma et al., 2022). DREB TFs are also involved in multiple abiotic stress processes, such as drought, salt, chilling, and heat. For instance, the suppression of *TaDTG6‐B* in wheat has been shown to diminish its drought tolerance (Mei et al., 2022). *RAP2.4* was known to be involved in response to low temperature in *Chrysanthemum*. Overexpression of *ClRAP2.4* has been observed to significantly influence the levels of reactive oxygen species (ROS) and malondialdehyde (MDA), thereby enhancing the cold tolerance (Ren et al., 2023). Furthermore, the TF *BaDBL1* could significantly upregulate the expression of stress response genes like *RD29A*, *RD29B*, *LEA*, and *ABI5*, thereby conferring osmotic and salt stress tolerances in transgenic *Arabidopsis* (Liang et al., 2021). *DREB2A* was also noted for its ability to activate the expression of *HsfA3*, which played a regulatory role in the heat stress response of *Arabidopsis* (Schramm et al., 2008).

As a pioneering tree species with well‐characterized genomic information, poplar is frequently utilized as a model tree for forest genetic research. To date, a total of 170 AP2/ERF members have been identified in poplar (Vahala et al., 2013). In our study, we pinpointed a *DREB* gene from *Populus alba* × *P. glandulosa*, named *PagERF021*, which could be induced under salt stress conditions. The gene expression pattern and protein characteristics indicated that it was involved in salt stress process. Furthermore, the *PagERF021*‐overexpressing plants were cultivated to perform salt stress tolerance and results showed that *PagERF021* gene played a positive regulatory role in the tolerance to salt stress in poplar. In conclusion, our research established the molecular function of *PagERF021* gene in response to salt stress, thereby providing genetic resources for molecular breeding programs focused on enhancing the salt tolerance of poplar tree.

## 2 MATERIALS AND METHODS

### 2.1 Plant materials

The hybrid 84k poplar (*Populus* *alba* × *P. glandulosa*) and *Nicotiana benthamiana* were used as material, which were cultivated in the State Key Laboratory of Northeast Forestry University, Harbin, Heilongjiang Province, China. The seedlings were cultured under greenhouse condition, 25°C with a 16:8 h light‐dark cycle. The various tissues were collected, including primary leaves (PL), mature leaves (ML), primary stems (PS), mature stems (MS), and roots [Correction added on October 4, 2024, after first online publication: “primary leaves” is change to “primary leaves (PL)” and “mature stems” is change to “mature stems (MS)”.]. Additionally, samples of roots, stems, and leaves treated with NaCl solution at 0, 6, 12, 24, and 48 h were also collected and immediately stored in a −80°C refrigerator.

### 2.2 Isolation and characterization of *PagERF021* gene

*PagERF021* gene was successfully cloned from 84 poplar and linked to pMD 19‐T vector (Takara). The obtained cDNA fragment was sequenced, and the amino acid composition and molecular weight of the encoded protein were analyzed using the ProtParam website (https://web.expasy.org/protparam/). Subsequently, multiple sequence alignment and phylogenetic tree analysis were conducted using the deduced amino acid sequences. The analysis was performed with the aid of Muscle and MEGA7 software, respectively (Kumar et al., 1994). The sequences included for comparison were XP_002325519.1 form *Populus trichocarpa*, XP_011005172.1 from *Populus euphratica*, AT1G71450.1 and AT1G33760.1 from *Arabidopsis thaliana*, XP_015616190.2 from *Oryza sativa*, LOC100815719 from *Glycine max*, and NP_0013592301 from *Zea mays*.

### 2.3 RNA isolation and qRT‐PCR analysis

Total RNA was isolated from various tissues using cetrimonium bromide (CTAB) method (Li et al., 2013). cDNA was synthesized using PrimeScript RT reagent Kit with gDNA Eraser (TaKaRa) according to manufacturer's instructions. Quantitative real‐time polymerase chain reaction (qRT‐PCR) was conducted on the Applied Biosystems 7500 Real‐Time PCR System. The amplification cycling conditions for amplification were performed according to manufacturer's instructions. *Actin* was used as internal reference and gene relative expression level was calculated by 2^−ΔΔCt^ (Regier & Frey, 2010). Each experiment was performed with three technical replicates. The sequences of the qRT‐PCR primers are listed in Supporting Information.

**Core Ideas**

- *PagERF021*, a DREB gene, could be induced by salt stress and predominantly expressed in the roots and stems.
- The PagERF021 protein was a nuclear localization protein with transcriptional activation activity.
- *PagERF021* gene could enhance salt tolerance by scavenging ROS and reducing cell membrane damage in poplar.

### 2.4 Plasmid construction

To construct the *PagERF021*‐green fluorescent protein (GFP) recombinant plasmid, the open reading frame of *PagERF021* gene was linked into pUC19‐GFP and pBI121‐GFP vectors using T4 DNA ligase (Takara), respectively. To generate pBI101‐pro*PagERF021*‐GUS (β‐glucuronidase) vector, the upstream promoter sequence of *PagERF021* gene was amplified and inserted into pBI101‐GUS with *Xba*I by homologous recombination. The recombinant plasmids, Pro*PagERF021*‐GUS and pBI121‐*PagERF021*‐GFP, were subsequently transformed into *Agrobacterium tumefaciens* GV3101 strains.

### 2.5 Subcellular localization of *PagERF021* protein

The pUC19‐GFP and pUC19‐*PagERF021‐*GFP recombinant plasmids were transformed into protoplasts from *Populus* *alba × P. glandulosa*, respectively. The method of protoplasts isolation was referred to the protocol described by F. H. Wu et al. (2009). The quantity of protoplasts was determined using a hemocytometer. The transformation of protoplasts was carried out using a polyethylene glycol‐mediated transient expression system. Furthermore, the *A*. *tumefaciens* strains with pBI121‐GFP and pBI121‐*PagERF021*‐GFP were injected into *N. benthamiana* leaves and incubated for 24–48 h. Subsequently, the GFP fluorescence was observed using a Zeiss LSM800 microscope.

### 2.6 Transcriptional activation analysis and Y1H assay

The full length of *PagERF021* gene was amplified and linked into pGBKT7 vector. Subsequently, *PagERF021* gene was truncated into three fragments, which also inserted into pGBKT7 vector. The pGBKT7 vector alone and pGBKT7‐53/pGADT7‐T were served as negative and positive controls, respectively. All constructs were transformed into yeast two‐hybrid (Y2H) strains and screened on SD/‐Trp and SD/‐Trp/‐His/‐Ade/‐X‐α‐gal solid medium for the interaction assay. The plates were incubated at 30°C for 3 days to identify positive interactions.

The oligonucleotide sequences containing three tandem repeats of the GCC‐box, DRE, and MYB‐core motif were synthesized. The fragments were then inserted into the pAbAi vector to create a bait vector. Subsequently, *PagERF021* gene was ligated into the pGADT7 vector to generate a pGADT7‐PagERF021 fusion vector. The bait vector was introduced into Y1H Gold yeast strain. The recombinant pGADT7‐PagERF021 plasmid was transformed into positive bait yeast, which was subsequently screened on SD/‐Leu and SD/‐Leu/‐AbA to identify interactions.

The MYB‐core element with three tandem repeats was cloned into the pCAMBIA1301 vector to form a reporter vector. Concurrently, the full‐length PagERF021 sequence was inserted into the pROK2 vector, resulting in the pROK2‐PagERF021 effector vector. Both the pCAMBIA1301‐MYB‐core reporter vector and pROK2‐PagERF021 effector vector were transformed into *A*. *tumefaciens* GV3101 strains. Subsequently, these strains were used for infiltration into tobacco leaves. As a control, the reporter vector was co‐transformed with an empty pROK2 vector.

### 2.7 Plant genetic transformation

Three‐week‐old poplar leaves were used for genetic transformation. The third to fifth leaves were submerged in a solution of *A*. *tumefaciens*. Following a 2‐day incubation period in darkness, the leaves were transferred to a screening medium. The medium contained 0.5 mg/L 6‐BA, 0.05 mg/L α‐naphthaleneacetic acid (NAA), 20 g/L sucrose, 5 g/L agar, and 40 mg/L kanamycin, supplemented with Woody Plant Medium. When resistant buds grew to 1 cm, the cuttings were cultured on the rooting medium including 0.05 mg/L indole‐3‐butyric acid, 0.02 mg/L NAA, 30 g/L sucrose, 5 g/L agar, 50 mg/L kanamycin, and 1/2 Murashige and Skoog medium. DNA and RNA from the rooted cuttings were extracted using CTAB method.

### 2.8 Salt stress treatment and physiological measurement

Two‐month‐old seedlings were treated with 200 mM NaCl solution for 5 days, with water as the control. Following the treatment, the height and fresh weight of the transgenic lines were measured. The third to fifth leaves were harvested for determination of physiological indices. The activities of peroxidase (POD), superoxide dismutase (SOD), and MDA were quantified using assay kits from Suzhou Comin Biotechnology, according to the manufacturer's protocol (X. Zhang et al., 2020). The chlorophyll content was determined using the method described by Vernon (1960).

### 2.9 Histochemical staining

One‐month‐old poplar seedlings were exposed to the 200 mM NaCl solution for 4 h, with water as the control treatment. Following the treatment, the leaves of each lines were harvested and these samples were immersed in solutions of nitroblue tetrazolium (NBT), 3,3′‐diaminobenzidine (DAB), and Evans Blue, respectively. The staining was carried out overnight at 37°C under dark conditions (K. Chen et al., 2019). Subsequently, the leaves were decolorized using absolute ethanol. The decolorized leaves were then observed and photographed to assess the staining patterns.

Three‐week‐old transgenic plants containing the pBI101‐pro*PagERF021*‐GUS construct and tobacco leaves were subjected to vacuum infiltration with a GUS staining solution. This solution contained 0.2 M phosphate buffer solution, 0.05 M K₃Fe(CN)₆, 0.5 M K₄Fe(CN)₆, 0.5 M ethylenediaminetetraacetic acid (EDTA), 2% Triton‐100, and 0.6% 5‐bromo‐4‐chloro‐3‐indolyl β‐D‐glucuronide. The vacuum infiltration was performed for 15 min. Subsequently, all samples were incubated at 37°C in the dark overnight to allow for the GUS reaction. After the incubation, the samples were decolorized and photographs were taken to observed the staining patterns.

### 2.10 Transcriptome analysis

The leaves from the overexpression of *PagERF021* transgenic lines and wild type lines were collected and promptly submitted for transcriptome sequencing on the Illumina platform at Genewiz company. Three biological replicates were performed for each group. RNA‐sequencing data were analyzed using the Hisat2 software to align the reads to the reference genome of *P. trichocarpa* with default parameters. The expression levels of the reference genes were quantified from the aligned reads. Differentially expressed genes (DEGs) were screened with two standards: *p*‐value ≤ 0.05 and |log_2_ fold change| ≥1. Gene ontology (GO) enrichment and visualization were performed using TBtools (C. Chen et al., 2020).

### 2.11 Statistical analysis

All experiments in the study were analyzed with three biological replicates, and the data were presented as mean ± standard deviation. Statistical significance was determined using *t*‐test (*p < 0.05, **p < 0.01) [Correction added on October 4, 2024, after first online publication: “was” is change to “were”].

## 3 RESULTS

### 3.1 Isolation and characterization of *PagERF021* gene

**FIGURE 1:**
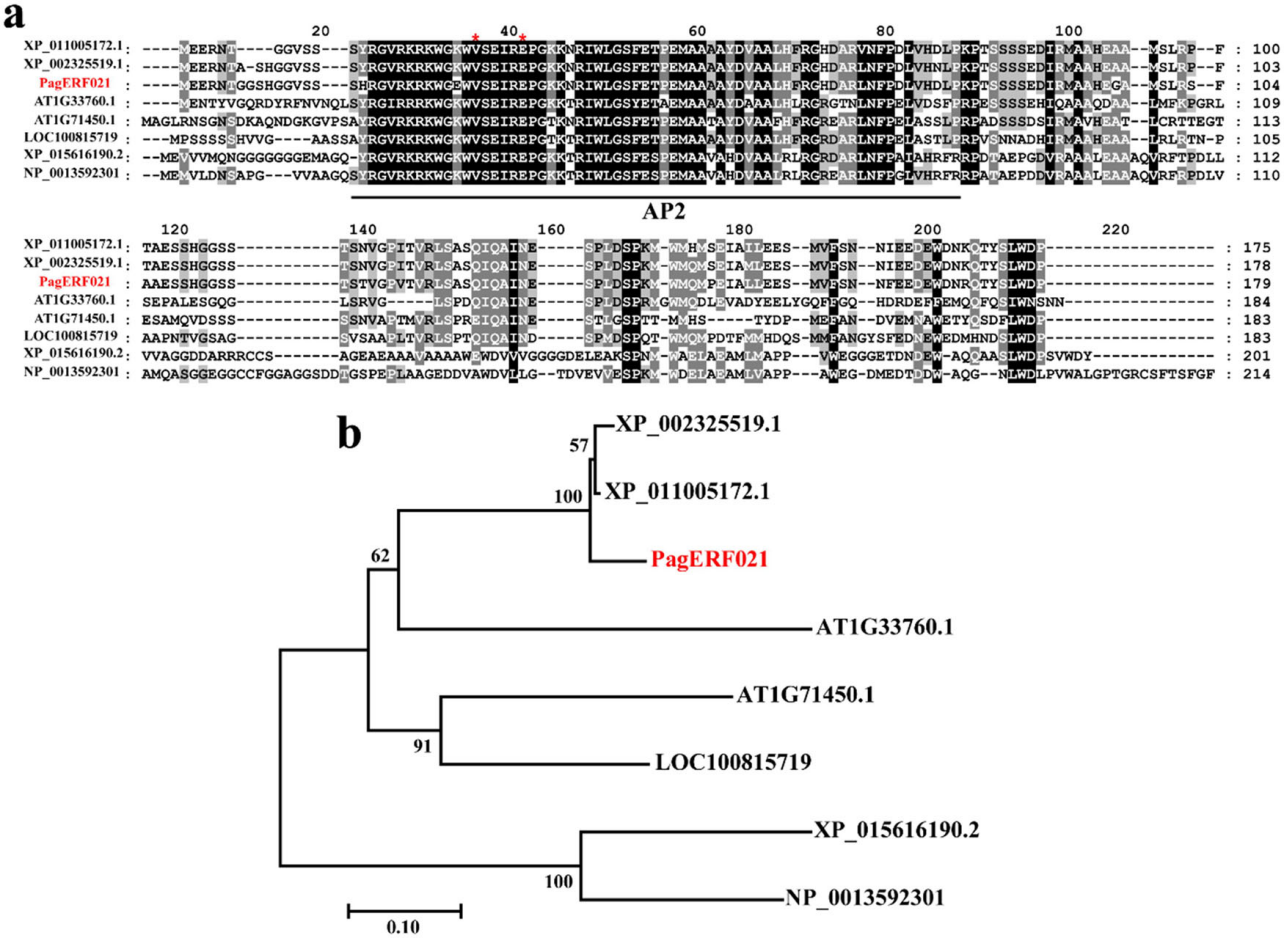
Sequence analysis of *PagERF021* protein. (a) Multiple sequence alignment between *PagERF021* protein and other homology proteins from different species. The red stars represent the 14th and 19th amino acids of proteins, respectively. The black line represents the AP2 domain. (b) Phylogenetic analysis between *PagERF02*1 protein and other homology proteins by MEGA 7 using neighbor‐joining method with a bootstrap value of 1000 replicates.

The *PagERF021* gene was 540 base pairs (bp), encoding a protein of 179 amino acids. The region from amino acids 16 to 79 encoded the typical AP2 domain, with the 14th and 19th amino acids being valine (Val) and glutamic acid (Glu), respectively (Figure 1a). The molecular weight of the PagERF021 protein was 19.93 kilodaltons (kDa). Multiple sequence alignment indicated PagERF021 protein shared 92.22% sequence identity with XP_011005172.1 from *P. euphratica*, 90.56% with XP_002325519.1 from *P. trichocarpa*, 62.64% with LOC100815719 from *G. max*, 55.68% with AT1G71450.1 and 52.98% with AT1G33760.1 from *A. thaliana*, 43.41% with XP_015616190.2 from *O. sativa*, and 37.99% with NP_0013592301 from *Z. mays*. Additionally, a phylogenetic analysis showed that PagERF021 protein shared the same clade with XP_011005172.1 and XP_002325519.1, and was also closely related to AT1G71450.1 (Figure 1b).

### 3.2 Expression pattern analysis of *PagERF021* gene under salt stress

**FIGURE 2:**
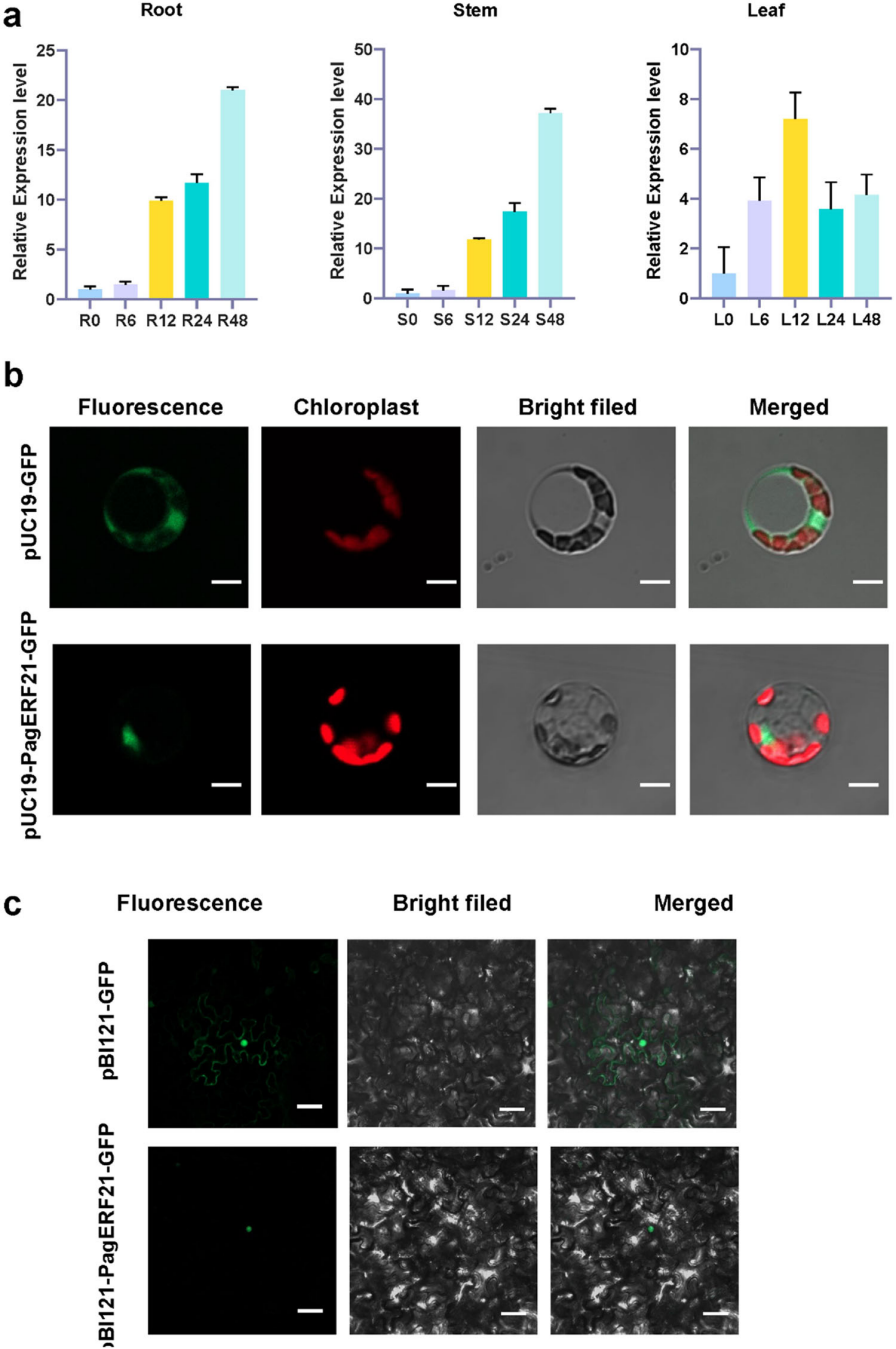
The expression pattern and subcellular localization of *PagERF021*. (a) The expression pattern of *PagERF021* gene in the roots, stems, and leaves under NaCl treatment. (b–c) Subcellular localization of *PagERF021* protein using poplar protoplast and tobacco system. Scale bar = 20 µm.

To detect whether *PagERF021* gene was induced by salt stress, qRT‐PCR was used to detect its expression pattern in roots, stems, and leaves under salt stress. The expression profiles of *PagERF021* gene exhibited similar trends, increasing to varying extents in response to salt stress (Figure 2a). In both roots and stems, the gene expression peaked at 48 h post‐treatment, reaching approximately 21.0‐fold and 37.2‐fold higher than under control conditions, respectively. In contrast, the highest expression level in leaves was observed at 12 h post‐treatment, with an approximately 7.2‐fold increase compared to the control.

### 3.3 Subcellular localization and transcriptional activation analysis

**FIGURE 3:**
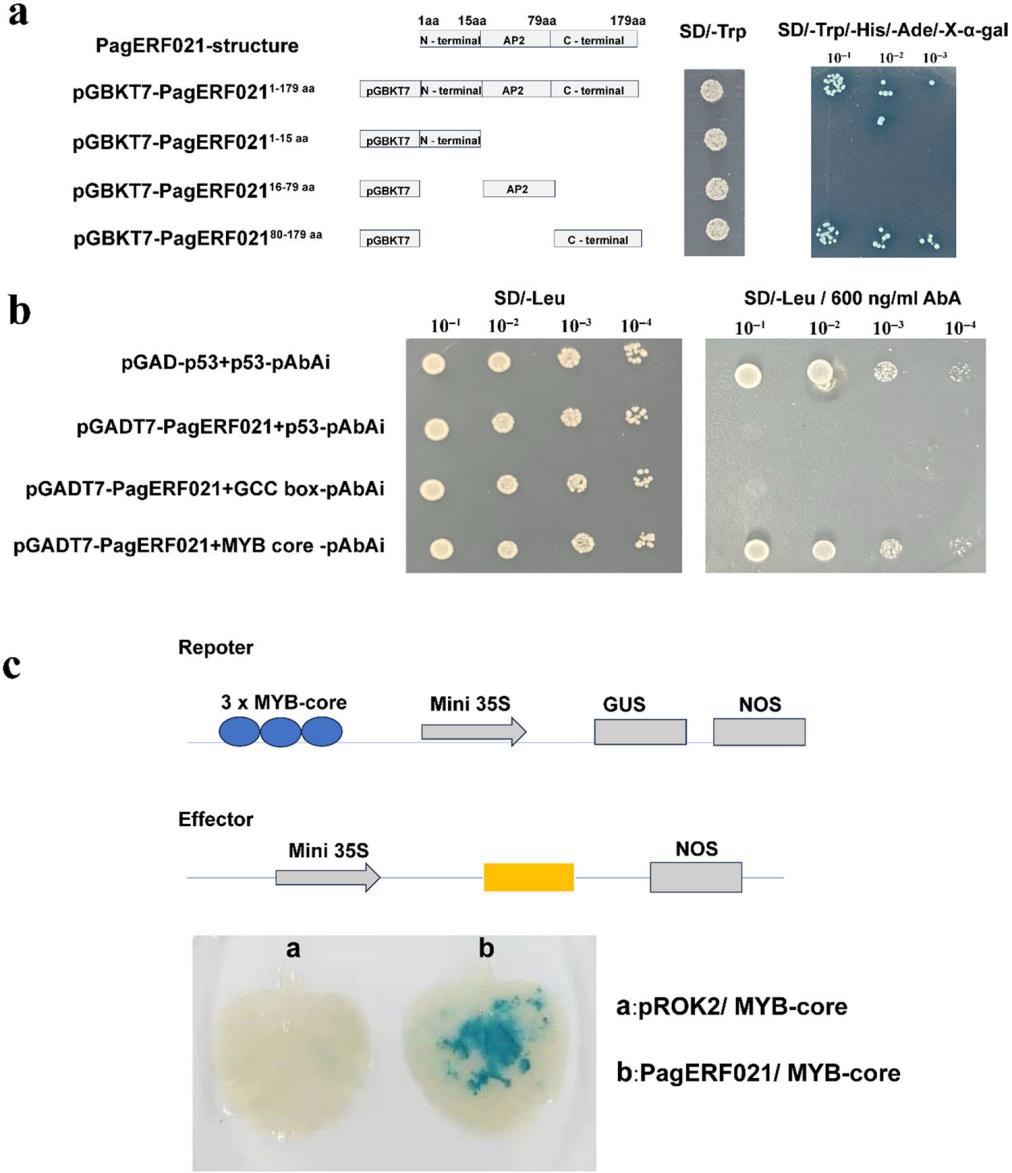
Transcriptional activation analysis and specific binding element of *PagERF021* protein. (a) Transcriptional activation analysis of *PagERF021* protein. (b) Y1H experiment about interaction between *PagERF021* protein and GCC‐box and MYB core element, respectively. (c) Schematic diagrams of the effector and reporter vectors used for co‐expression in tobacco and β‐glucuronidase (GUS) staining of tobacco leaves. NOS, nitric oxide synthase.

We investigated the subcellular location of PagERF021 protein using both poplar protoplasts and tobacco system. The positive control proteins, pUC19‐GFP and pBI121‐GFP, were expressed throughout the cell, while the fusion protein PagERF021‐GFP was only specifically localized to the nucleus (Figure 2b,c). Furthermore, the transcriptional activation activity of PagERF021 protein was verified by the yeast two‐hybrid system. As shown in Figure S1, all the transformants could grow on SD/‐Trp medium, the positive control and PagERF021‐BD could grow on SD/‐Trp/‐His/‐Ade medium and exhibited a blue coloration with X‐α‐gal, while the negative control did not grow on this medium. To confirm the transcriptional activation region of PagERF021 protein, we carried out a yeast transformation experiment. The protein was divided into three segments: pGBKT7‐PagERF021^1‐15 aa^, pGBKT7‐PagERF021^16‐79 aa^, and pGBKT7‐PagERF021^80‐179 aa^, with the flanking of AP2 domain. Only pGBKT7‐PagERF021^80‐179 aa^ could grow and turned blue on the SD/‐Trp/‐His/‐Ade/‐X‐α‐gal medium, while other fragments could not grow on this medium (Figure 3a). These results showed that the transcriptional activation region of PagERF021 protein was located within the C‐terminal region, specifically within the 80–179 amino acid segment [Correction added on October 4, 2024, after first online publication: “These results showed that *PagERF021* protein was located within the C‐terminal region, specifically within the 80–179 amino acid segment.” is changed to “These results showed that the transcriptional activation region of PagERF021 protein was located within the C‐terminal region, specifically within the 80–179 amino acid segment”.] .

### 3.4 Specific binding of *PagERF021* protein to MYB‐core element

We utilized a yeast one‐hybrid (Y1H) system to ascertain the interaction between the PagERF021 protein and three distinct elements. The DRE element was found to support normal yeast growth on SD/‐Leu/1000 ng/mL AbA solid medium, indicating it was not a suitable target for this system. Consequently, we focused further studies on the GCC‐box and MYB‐core elements. All co‐transformants could grow normally on SD/‐Leu medium. Notably, both the positive control and the transformants harboring the MYB‐core element along with PagERF021 could grow normally on SD/‐Leu/ 600 ng/mL AbA medium. In contrast, the negative control and those co‐transformed with the GCC box and PagERF021 failed to grow (Figure 3b). Furthermore, to substantiate our findings, we conducted transient transformations in tobacco. As shown in Figure 3c, the tobacco leaves displayed a blue coloration when co‐transformed with PagERF021 and the MYB‐core element, while the leaves of co‐transformed with the proke II and the MYB‐core element lacked color change. Collectively, these results implied that PagERF021 protein could specifically bind to the MYB‐core element.

### 3.5 Promoter sequence analysis of *PagERF021* gene

**FIGURE 4:**
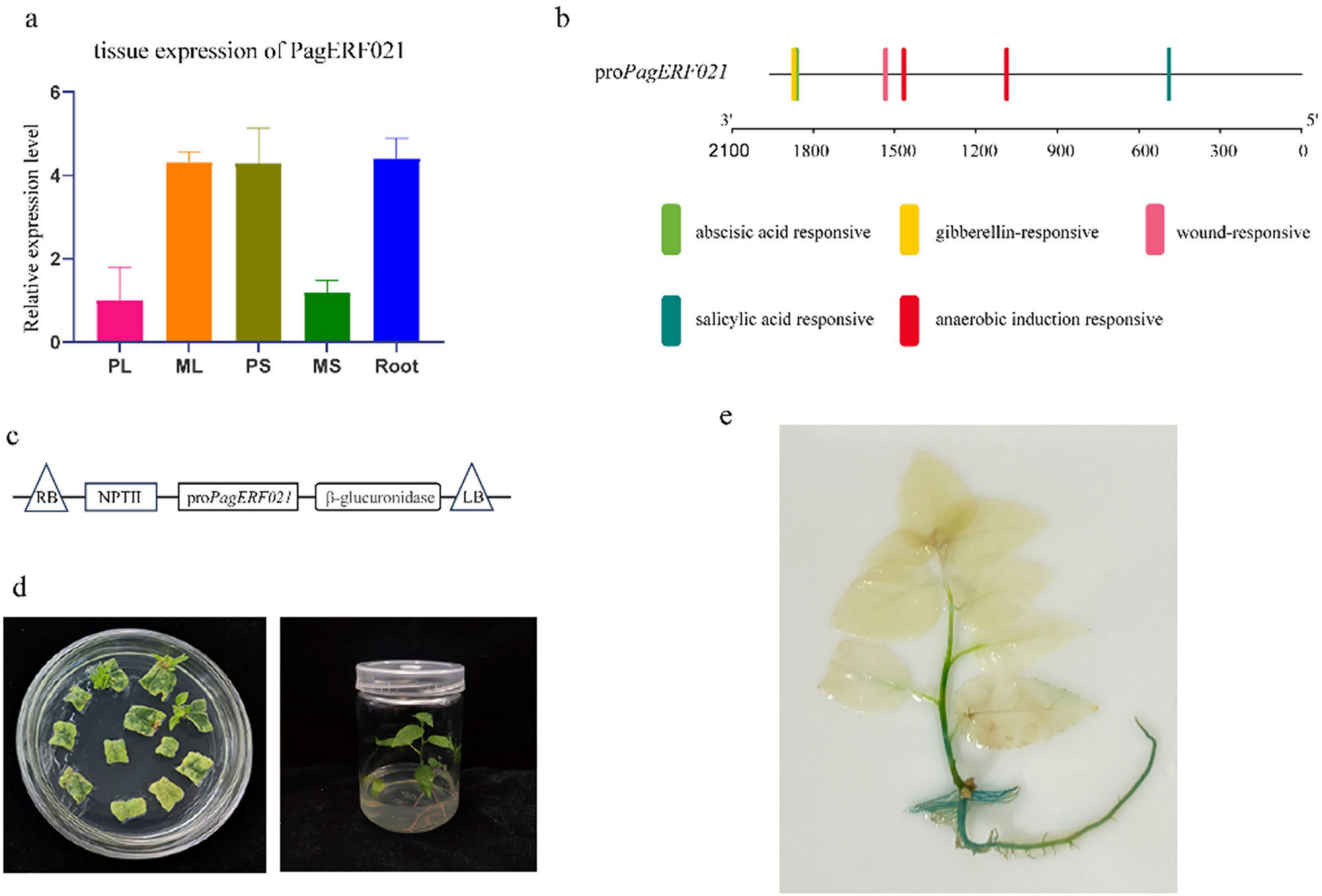
The sequence analysis of *PagERF021* promoter. (a) The expression of *PagERF021* gene in different tissues, including primary leaves (PL), mature leaves (ML), primary stems (PS), mature stems (MS), and roots. (b) The motif analysis of *PagERF021* promoter. (c) The scheme of pBI101‐pro*PagERF021*‐GUS recombinant expression vector. (d) Acquisition of pro*PagERF021*‐overexpression lines. (e) The GUS staining of pro*PagERF021*‐overexpression lines. GUS, β‐glucuronidase.

The tissue‐specific expression of *PagERF021* gene was assessed using qRT‐PCR. The results showed that *PagERF021* gene exhibited high levels of expression in roots, ML, and PS (Figure 4a). To further delineate the in vivo expression pattern of the *PagERF021* gene, the 1962 bp upstream sequence of this gene was cloned into GUS expression vector, yielding transgenic lines harboring the pro*PagERF021*‐GUS construct (Figure 4c,d). Analysis of the promoter sequence for *cis*‐acting elements using PlantCARE (http://bioinformatics.psb.ugent.be/webtools/plantcare/html/) revealed the presence of several pivotal elements, including the anaerobic induction responsive element, ABA responsive element, GA‐responsive GARE‐motif, wound‐responsive motif, and salicylic acid responsive element (Figure 4b). GUS staining of the pro*PagERF021*‐GUS plants indicated that the promoter sequence of *PagERF021* gene was capable of driving GUS gene expression, with intense staining observed in roots and stems, and comparatively weaker staining in leaves.

### 3.6 Plant traits of PagERF021 overexpression lines

**FIGURE 5:**
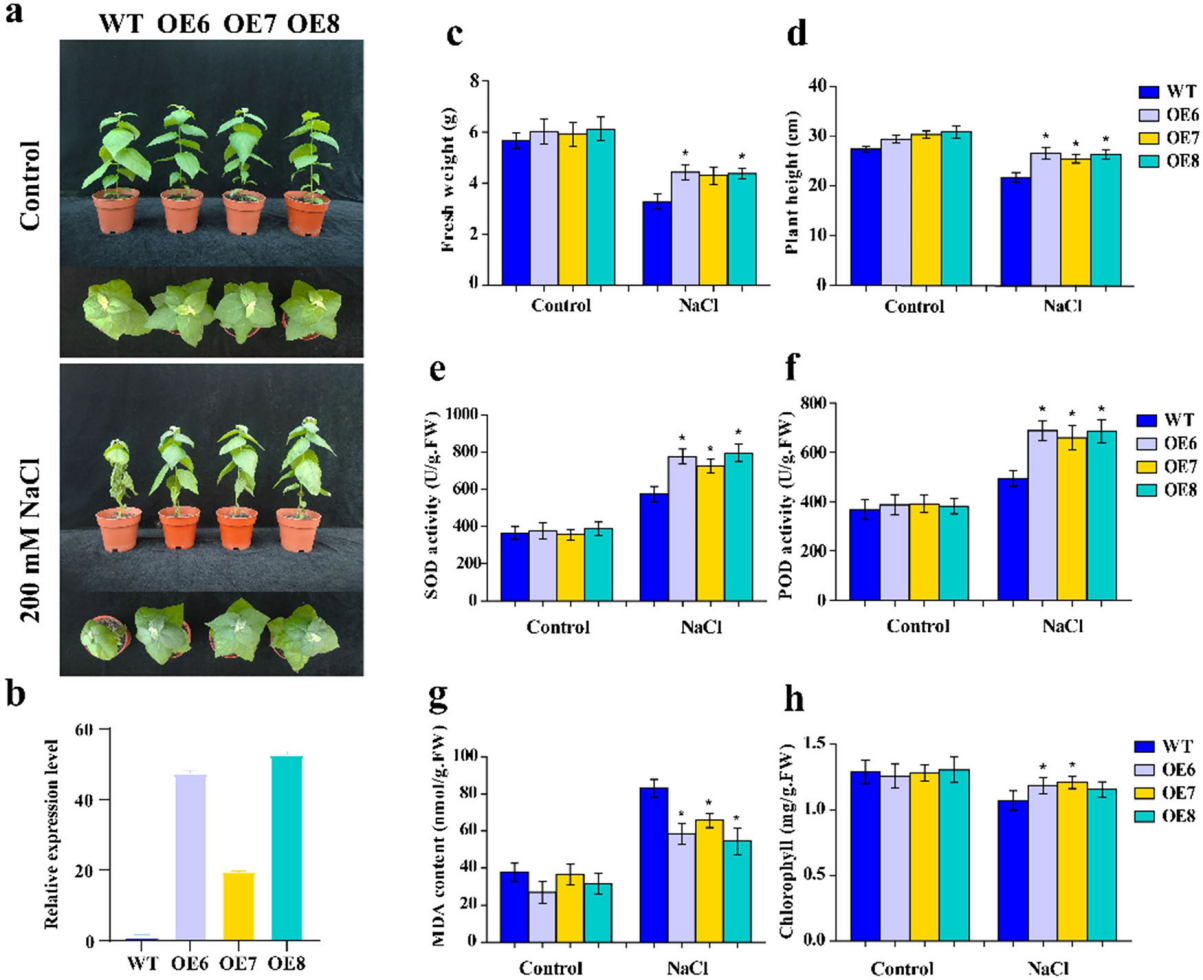
Determination of physiological indices of *PagERF021*‐overexpression lines under salt stress. (a) The growth state of *PagERF021*‐overexpression and wild type plants. (b) The expression level of *PagERF021*‐overexpression lines. (c–f) Physiological indicators of *PagERF021*‐overexpression lines. Error bars indicate mean ± SD. Asterisks indicate significant differences (*t‐*test, *p < 0.05; **p < 0.01).

In the study, three overexpression lines of *PagERF021* (OE6, OE7, and OE8) were generated by leaf disk method (Gallois & Marinho, 1995), and the expression of the gene in each line was detected using gene‐specific primers detailed in Table S1. The relative expression levels of *PagERF021* in OE6, OE7, and OE8 was ∼19.4‐ to 52.5‐fold than that of wild type lines (Figure 5b). Under normal growth condition, the developmental status of the transgenic lines paralleled that of wild type lines. However, under 200 mM salt stress for 7 days, wild type plants exhibited signs of stress such as leaf wilting and drooping, while the transgenic plants showed resilience with no apparent growth inhibition. The plant height and fresh weight of the transgenic lines was 17.2%–22.3% and 31.0%–34.8% greater than those of wild type lines (Figure 5a,c,d). Physiological indices, including SOD and POD activities, as well as MDA content and chlorophyll levels, were measured. Under normal conditions, no significant differences in these physiological indices were observed between the transgenic and wild type plants. In contrast, under salt stress, the activities of SOD and POD in the transgenic lines were 26.4%–38.5% and 33.4%–39.1% higher than those of wild type poplars, respectively. Concurrently, the content of MDA was 21.0%–34.6% lower, and the content of chlorophyll was 7.9%–13.1% higher in the transgenic lines compared to those of wild type poplars (Figure 5e–h).

**FIGURE 6:**
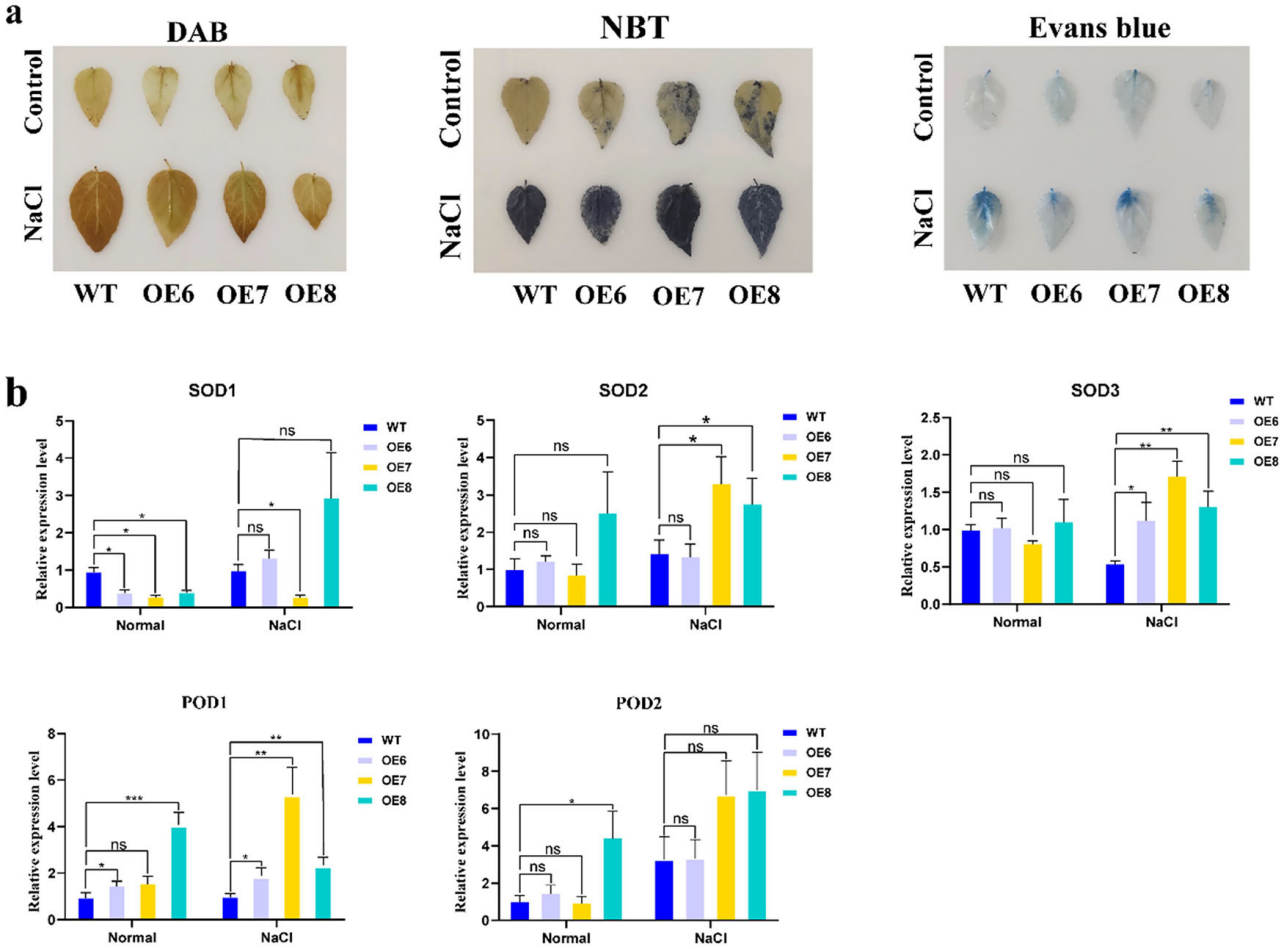
Nitroblue tetrazolium (NBT), 3,3'‐diaminobenzidine (DAB), and Evans blue staining and the expression level of peroxidase (POD) and superoxide dismutase (SOD)‐related genes in *PagERF021*‐overexpression lines. (a) NBT, DAB, and Evans blue staining. (b) The expression level of POD and SOD‐related genes. Asterisks indicate significant differences (*t‐*test, *p < 0.05; **p < 0.01).

Moreover, histological stainings with DAB, NBT, and Evans blue were performed on leaf samples [Correction added on October 4, 2024, after first online publication: “histological staining” to “histological stainings” and “was” is changed to “were”.]. Under normal conditions, similar staining intensities were observed across all plant lines. However, upon salt stress, the transgenic lines displayed reduced staining intensity and a smaller stained area, as represented in Figure 6a. Additionally, the expression levels of genes related to POD and SOD activities were also detected. Under normal condition, the expression levels of *SOD*‐related genes did not significantly differ among all plants. Notably, in the OE8 line, the expression levels of *POD1* and *POD2* were significantly elevated compared to wild type lines. Upon NaCl treatment, the expression levels of *SOD* and *POD*‐related genes were upregulated in all the transgenic lines, with *SOD2*, *SOD3*, and *POD1* genes showing particularly significant increase, as demonstrated in Figure 6b. The collective results indicated that the overexpression of *PagERF021* gene could improve the salt tolerance of transgenic poplar plants.

### 3.7 Transcriptome data analysis

**FIGURE 7:**
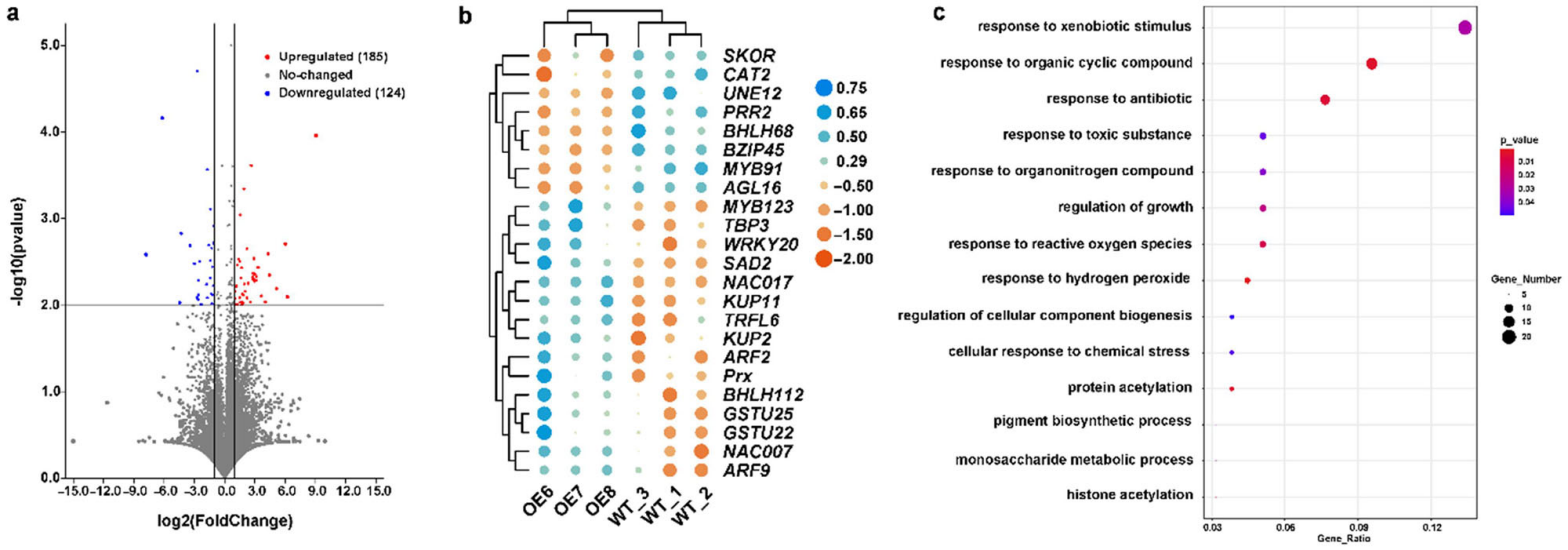
Transcriptome analysis of *PagERF021* transgenic poplar compared to wild type poplars. (a) Volcano plot analysis. (b) Heatmap of stress‐related genes. (c) Gene ontology (GO) enrichment analysis of differentially expressed genes (DEGs).

In order to further explore the regulatory mechanism of *PagERF02*1 gene, transcriptome sequencing was conducted on *PagERF021* transgenic poplar compared to wild type poplars. This analysis revealed a total of 309 DEGs, including 185 upregulated genes and 124 downregulated genes (Figure 7a) [Correction added on October 4, 2024, after first online publication: “121” is change to “124”.]. A significant number of these DEGs are associated with stress responses in PagERF021‐transgenic plants. These include genes encoding glutathione S‐transferases (*GST*s), peroxiredoxins (*Prx*), catalase 2, S‐adenosylmethionine decarboxylase 2 (*SAD2*), *bZIP* *45*, MYB TFs, basic helix‐loop‐helix TFs, NAC TFs (*NAC007*, *NAC017*), auxin response factors (*ARF*s), *WRKY20*, and *AGL16* (Figure 7b). Notably, several DEGs were involved in the uptake and transport of potassium, such as potassium uptake permease 2 (*KUP2*), *KUP11*, and *SKOR* (Figure 7b). Detailed information can be referred to Table S2.

GO enrichment analysis indicated significant enrichment in categories related to the response to xenobiotic stimuli (GO:0009410), response to organic cyclic compound (GO:0014070), response to antibiotic (GO:0046677), regulation of growth (GO:0040008), response to organonitrogen compound (GO:0010243), and so on were significantly enriched. Furthermore, it is noteworthy that terms related to ROS were also significantly enriched, including response to toxic substances (GO:0009636), response to hydrogen peroxide (GO:0042542), response to ROS (GO:0000302), and cellular response to chemical stress (GO:0062197) (Figure 7c). The findings demonstrated the function of *PagERF021* gene in modulating the transcriptome, particularly in response to stress [Correction added on October 4, 2024, after first online publication: text “…and the regulation of growth and ROS homeostasis” is deleted.].

## 4 DISCUSSION

ERF family is one of the plant‐specific TFs family that have been extensively analyzed and identified in *Arabidopsis*, rice, and maize (Qi et al., 2023; Rashid et al., 2012; Sakuma et al., 2002). To date, a total of 170 AP2/ERF members have been identified in *P*. *trichocarpa*. Among these, there were 77 members of the DREB subfamilies, which were divided into five groups (Vahala et al., 2013). An increasing researches indicated that DREB members were involved in the response to multiple stresses and acted as key regulators in plant stress resistance (Agarwal et al., 2006; Liu et al., 2000) [Correction added on October 4, 2024, after first online publication: “are” is changed to “were”.]. In our studies, we identified a member of DREB A4 subgroup, *PagERF021* gene, which was found to be highly induced by salt stress. To explore its function, the gene was cloned and was found to encode a protein of 179 amino acids (Figure 1a). A phylogenetic analysis revealed that PagERF021 shared a cluster with AT1G71450.1 from *A. thaliana* (Figure 1b), whose expression was associated with ethylene insensitivity and the suppression of EDF1/2/3/4 and abscission‐associated genes (W. H. Chen et al., 2015). We aimed to explore the role of the *PagERF021* gene in abiotic stress responses, an area that remained largely uncharted in poplar.

Nuclear localization and transcriptional activation activity are pivotal attributes of TFs. *PagERF021* protein was found to be exclusively localized to the nucleus (Figure 2b,c), and its transcriptional activation activity region was mapped to the C‐terminal region, specifically 80–179 amino acids (Figure 3a). As a transcriptional activator, *PagERF021* protein modulated gene expression within the nucleus. Recent reports have elucidated the diverse molecular roles of the non‐AP2 conserved regions of ERF family members. For instance, *PpERF98‐1* and *PpERF98‐2* interacted with *PpERF1‐1/2*, promoting to peach susceptibility to *Lasiodiplodia theobromae* (D. Zhang et al., 2023). Additionally, *HvRAF*, characterized by a critical hemopexin domain signature‐like sequence at its C‐terminus, has been shown to enhance pathogen resistance and salt tolerance when overexpressed in *Arabidopsis* (J. Jung et al., 2007). These findings parallel our results, suggesting that PagERF021 protein may participate in multiple molecular processes, potentially through complexes formation with other protein.

ERF TFs participate in various biological processes, primarily through their specific binding to GCC‐box and DRE elements in the promoters of target gene (Sakuma et al., 2002). For example, the *Arabidopsis AtERF71/HRE2* functioned as a transcriptional activator via the *cis*‐acting GCC‐box or DRE/CRT element and was involved in root development by modulating root cell expansion (S. Y. Lee et al., 2015). *TINY*, a DREB‐like TF, mediated signaling pathways by binding to the DRE and ethylene response element in *A. thaliana* (Sun et al., 2008). Additionally, as a transcriptional activator, *DREB2c* can could the DRE element of the tolerance‐related phytocystatin 4 (*atCYS4*) gene, thereby enhancing the heat tolerance of *Arabidopsis* (Je et al., 2014) [Correction added on October 4, 2024, after first online publication: “can” is change to “could”.]. Recent studies have indicated that ERF family could also interact with other motifs to regulate gene expression. For example, *PeDREB28* could bind to the CE1‐CRE element in the promoter of pyrabactin‐resistance‐like gene (*DlaPYL3*), which was a homolog of ABA receptor in *Arabidopsis*, and activated its expression (Hu et al., 2023) [Correction added on October 4, 2024, after first online publication: “activates” is change to “activated”.]. Similarly, the expression of *BpERF13* gene was induced by low temperature stress in *Betula platyphylla*, and the expression levels of *POD‐* and *SOD*‐related genes were activated by binding with MYB‐core element, improving the cold tolerance of transgenic plants (Lv et al., 2020). In the study, *PagERF021* protein also could specifically bind to MYB‐core element, as demonstrated by yeast one‐hybrid (Y1H) and transient transformation of tobacco (Figure 3b,c). Furthermore, the overexpression of *PagERF021* transgenic poplars also influenced the expression levels of *SOD‐* and *POD*‐related genes (Figure 6b). *PagERF021* gene might also share similar functions with *BpERF13* and participated in analogous regulatory pathway [Correction added on October 4, 2024, after first online publication: “participate” is change to “participated”.].

When faced with salt stress, plants give rise to osmotic stress and ion toxicity, leading to the accumulation of excessive ROS, which can cause oxidative damage to DNA, cell membrane, and lipids (Gupta et al., 2015). To mitigate this oxidative damage, plants activate enzymes and nonenzymatic antioxidants to scavenge ROS (Habib et al., 2016). POD and SOD are key enzymatic antioxidants that help maintain ROS homeostasis in plants (Das & Roychoudhury, 2014; Ahmad et al., 2008). In our study, under normal conditions, there was no significant difference in the activities of POD and SOD among all plants. However, upon salt stress treatment, the activities of POD and SOD in the *PagERF021*‐overexpressing lines were significantly higher than those of wild type plants (Figure 5e,f). And the expression of *SOD‐* and *POD*‐related genes was consistent with these enzyme activities (Figure 6b). Additionally, DAB and NBT staining showed the lower degree of staining in the *PagERF021*‐overexpressing lines under salt stress (Figure 6a). MDA, a product of lipid peroxidation, serves as a marker for membrane damage (Jadoon & Malik, 2017). The MDA content of *PagERF021*‐overexpressing lines was only 21.0%–34.6% of wild type plants (Figure 5g). Evans blue staining also further indicated that the cell membrane damage in the *PagERF021*‐overexpressing lines was weaker than those of wild type lines.

Furthermore, our transcriptome analysis revealed a total of 309 DEGs, impacting the expression of various stress‐related genes such as *WRKY20*, *NAC17*, *SAD2*, *AGL16*, *KUP* (‐2, ‐11), *SKOR*, *GST* (‐22, ‐25), and *Prx (*Figure 7b *). WRKY20*, a member of the WRKY TF, was involved in plant immune and abiotic stress responses (Luo et al., 2013; Yan et al., 2017). Overexpression of *GsWRKY20* from *Glycine soja* has been shown to enhance the drought and salt tolerance in transgenic alfalfa (Tang et al., 2014). *OsNAC17* was considered as a candidate gene for drought resistance in rice. Its overexpression was reported to positively regulate the expression of multiple lignin biosynthesis genes (*4CL*, *CAD*, *LAC*, and *PAL*), leading to increase lignin accumulation and improve drought resistance in rice (S. E. Jung et al., 2022). *LbSAD2*, a homologous gene of *SUPER SENSITIVE TO ABA AND DROUGHT2*, has been demonstrated to reduce root hair development, Na^+^ accumulation, and ABA sensitivity, thereby enhancing salt tolerance in *Arabidopsis* (Xu et al., 2021). MADS TF *AGL16* acted a negative regulator of stress responses and directly inhibited the expression of the expression of stress‐related genes such as *HKT1;1*, *HsfA6a*, and *MYB102* genes, reducing the sensitivity to salt stress in *Arabidopsis* (Zhao et al., 2021). Potassium (K^+^) was the most abundant cation in higher plants and was crucial for plant nutrition, growth, tropism, enzyme homeostasis, and osmotic regulation (Nieves‐Cordones et al., 2016; Shabala & Pottosin, 2014). In *Arabidopsis*, *AtKUP*s (1, 2, 4, 5–7, 10, and 11) have been implicated in K^+^ transport, indicating that these KUP proteins played an important role in K^+^ transport (Ahn et al., 2004) [Correction added on October 4, 2024, after first online publication: “play” is changed to “played”.]. Overexpression of *AtKUP2* has been shown to maintain a higher K^+^/Na^+^ ratio, improving salt tolerance (Rajappa et al., 2020). The SKOR K^+^ channel, which contained a single cysteine residue critical for ROS sensitivity, has been identified as important for K^+^ transport and ROS signaling (Garcia‐Mata et al., 2010) [Correction added on October 4, 2024, after first online publication: “contain” is changed to “contained”.]. The roles of MYB TFs in abiotic stress have not been extensively reported, while *CaPPR2* has been confirmed to regulate drought and salt tolerance in *Capsicum annuum* (Lim et al., 2021) [Correction added on October 4, 2024, after first online publication: text “CaPPR2 has been confirmed to regulate drought and salt tolerance in C. annuum (Lim et al., 2021)” is deleted.]. The significant changes in the expression levels of these genes in *PagERF021*‐overexpressing plants suggest that *PagERF021* gene might enhance plant salt tolerance by interacting with these stress‐related genes.

## 5 CONCLUSION

In our study, we successfully cloned *PagERF021 gene*, a DREB member from 84K poplar, demonstrating its inducibility by salt stress across various tissues. Expression analysis revealed high levels of *PagERF021* gene in the roots and stems. The protein was localized in the nucleus, with its transcriptional activation region situated at the C‐terminal [Correction added on October 4, 2024, after first online publication: “domain” is changed to “region”.]. Furthermore, we found that the PagERF021 protein was capable of binding to the MYB‐core element. The overexpression of *PagERF021* transgenic lines enhanced the tolerance of salt stress in poplar in terms of activities of POD and SOD, chlorophyll content, and MDA levels. Transcriptome analysis highlighted significant enrichment of ROS‐related terms, suggesting a role for *PagERF021* gene in ROS scavenging. These findings suggested that *PagERF021* gene played a positive regulatory role in the tolerance to salt stress in poplar, providing new evidence for the role of ERF genes in plant responses to abiotic stress and offering feasible suggestions for the future construction of the ERF family regulatory network.

## AUTHOR CONTRIBUTIONS

**Gaofeng Fan**: Conceptualization; data curation; formal analysis; methodology; software; supervision; validation; visualization; writing—original draft; writing—review and editing. **Yuan Gao**: Data curation; investigation; methodology; validation. **Xinyue Wu**: Data curation; investigation. **Yingying Yu**: Data curation; investigation. **Wenjing Yao**: Investigation; methodology; writing—review and editing. **JiaHui Jiang**: Conceptualization; data curation; validation; visualization. **Huanzhen Liu**: Funding acquisition; project administration **Tingbo Jiang**: Data curation; funding acquisition; investigation; project administration; supervision; writing—review and editing.

## CONFLICT OF INTEREST STATEMENT

The authors declare no conflicts of interest.

## Supporting information




**Table S1**. Primer information
**Fig. S1**. Transcriptional activation analysis of PagERF021 protein
**Table S2**. Stress‐related DEGs information

## Data Availability

All data generated or analyzed during this study are included in this published article and information files. The raw sequencing data used during this study have been deposited in the NCBI's SRA with the accession number PRJNA1067173.
