# Supplementary material for: Functional analysis of PagERF021 gene in salt stress tolerance in Populus alba × P. glandulosa
Source: Plant Genome. 2024 Oct 16;17(4):e20521. doi: 10.1002/tpg2.20521 (PMC11628909; doi:10.1002/tpg2.20521)
Supplement: Supplementary file 1 [file TPG2-17-e20521-s001.docx]

**Primer information**

| **function** | **primer_name** | **primer_sequence(5'-3')** |
| --- | --- | --- |
| **PagERF021_gene_clone** | **PagERF021_F** | **ATGGAAGAGAGAAACACTGG** |
|  | **PagERF021-R** | **AGGATCCCAAAGAGAATATG** |
| **PagERF021_121_GFP** | **PagERF021_121_GFP_F**  **PagERF021_121_GFP_R** | **CTCTAGAATGGAAGAGAGAAACACTGG**  **CGTCGACAGGATCCCAAAGAGAATATG** |
| **PagERF021_promoter_clone** | **PagERF021_pro_F**  **PagERF021_pro_R** | **CAAGCTTTTGGTTAGTGAGCACTTTAA**  **CTCTAGATAATTCCCACTAAATTAAGTGAAG** |
| **PagERF021_pUC19** | **PagERF021_pUC19_F** | **TCTAGAATGGAAGAGAGAAACACTGG** |
|  | **PagERF021_pUC19_R** | **GTCGACAGGATCCCAAAGAGAATATG** |
| **Y2H_experiment** | **PagERF021_BD-F**  **PagERF021-BD-R**  **PagERF021_BD-F**  **pGBKT7-PagERF021^1-15aa^-R pGBKT7-PagERF021^16_79aa^_F pGBKT7-PagERF021^16_79aa^_R pGBKT7-PagERF021^80-179aa^_F PagERF021-BD-R** | **CCATATGATGGAAGAGAGAAACACTGG GGAATTCCTAAGGATCCCAAAGAGAAT CCATATGATGGAAGAGAGAAACACTGG GGAATTCAGAACTCACACCACCATGGC CCATATGAGCCATAGAGGGGTGAG GGAATTCTAGATCATGAACCAAGTCA CCATATGCCAAAGCCAACAAGCTC GGAATTCCTAAGGATCCCAAAGAGAAT** |
| **qRT-pcr** | **PagERF021_qRT_F**  **PagERF021_qRT_R**  **SOD1-F**  **SOD1-R**  **SOD1-F**  **SOD1-R**  **SOD4-F**  **SOD4-R**  **POD1-F**  **POD1-R**  **POD2-F**  **POD2-R**  **Actin-F**  **Actin-R** | **CTACCAAAGCCAACAAGC**  **ACGTTGGAGGTAGAGCTG**  **CTAATGTTGAAGGCGTCGTC**  **ACGCATCCATTTGTTGTGTC GCCTTGCCTGAGATACTTAC GCTTCAGTCATAGTCTTCAC GAATCTCACTCCTGTCCAAG CAACACCAAGTAATGGAACC GTCTTATGCTGACTTCTACC AATCCAGAACGCTCCTTGTG GGTTCTGTGCTTCTCGACTC TGTGCCATCTCTTCTTCCAG ACCCTCCAATCCAGACACTG TTGCTGACCGTATGAGCAAG** |

**Table S1. Primer information**

**Fig. S1. Transcriptional activation analysis of PagERF021 protein**

**Stress-related DEGs information**

| **annotation** | **gene** | **poplar_ID** | **Ath_ID** | **log2FC** | **P_value** |
| --- | --- | --- | --- | --- | --- |
| **MYB transcriptional factor** | **MYB91** | **Potri.004G102600** | **AT2G37630.1** | **-3.115477217** | **0.010030893** |
|  | **TRFL6** | **Potri.001G170200** | **AT1G72650.2** | **2.281570357** | **0.035479188** |
|  | **MYB123** | **Potri.006G221500** | **AT5G35550.1** | **2.974733241** | **0.037299415** |
|  | **PRR2** | **Potri.001G146200** | **AT4G18020.1** | **-2.165961551** | **0.002398019** |
|  | **TBP3** | **Potri.007G005000** | **AT5G67580.1** | **3.440572591** | **0.028747197** |
| **bHLH transcriptional factor** | **BHLH112** | **Potri.004G029100** | **AT1G61660.1** | **2.12495827** | **0.013545409** |
|  | **UNE12** | **Potri.013G041000** | **AT4G02590.1** | **-3.258734268** | **0.032677923** |
|  | **BHLH68** | **Potri.003G051600** | **AT4G29100.1** | **-5.371558863** | **0.010736951** |
| **NAC transcriptional factor** | **NAC017** | **Potri.005G200100** | **AT1G34190.1** | **2.186449677** | **0.001276674** |
|  | **NAC007** | **Potri.003G113000** | **AT1G12260.1** | **2.029747343** | **0.006833877** |
| **Auxin response factor** | **ARF2** | **Potri.003G163600** | **AT5G62000.1** | **2.555215157** | **0.016716609** |
|  | **ARF9** | **Potri.001G088600** | **AT4G23980.1** | **2.499097871** | **0.01731701** |
| **WRKY transcription factor** | **WRKY20** | **Potri.001G361600** | **AT4G26640.2** | **2.144389909** | **0.024508179** |
| **MADS-box transcription factor** | **AGL16** | **Potri.003G170000** | **AT3G57230.1** | **-4.528779665** | **0.000259878** |
| **bZIP transcription factor** | **BZIP45** | **Potri.001G029800** | **AT3G12250.1** | **-4.896875001** | **0.000376429** |
| **K+ uptake permease 11** | **KUP11** | **Potri.003G109800** | **AT2G35060.1** | **3.321928095** | **0.003686964** |
| **potassium transporter 2** | **KUP2** | **Potri.019G056500** | **AT2G40540.1** | **2.355292914** | **0.008703497** |
| **STELAR K+ outward rectifier** | **SKOR** | **Potri.012G043000** | **AT3G02850.1** | **1.702369254** | **0.048778694** |
| **glutathione S-transferase TAU** | **GSTU25** | **Potri.019G130500** | **AT1G17180.1** | **2.560714954** | **0.021971868** |
|  | **GSTU22** | **Potri.019G130600** | **AT1G78340.1** | **2.263034406** | **0.048742093** |
| **Peroxidase superfamily protein** | **Prx** | **Potri.007G132800** | **AT5G24070.1** | **2.115477217** | **0.020968228** |
| **catalase 2** | **CAT2** | **Potri.002G009800** | **AT4G35090.1** | **-2.04075175** | **0.022777155** |
| **ARM repeat superfamily protein** | **SAD2** | **Potri.014G149600** | **AT2G31660.1** | **5.151309323** | **0.006408914** |

**Table S2. Stress-related DEGs information**
